# Gamma rhythms in the visual cortex: functions and mechanisms

**DOI:** 10.1007/s11571-021-09767-x

**Published:** 2021-12-22

**Authors:** Chuanliang Han, Robert Shapley, Dajun Xing

**Affiliations:** 1 State Key Laboratory of Cognitive Neuroscience and Learning & IDG/McGovern Institute for Brain Research, Beijing Normal University, Beijing, 100875 China; 2 Center for Neural Science, New York University, New York, NY USA

**Keywords:** Gamma rhythm, Visual cortex, Neural network, Dynamic system, Computational Model

## Abstract

Gamma-band activity, peaking around 30–100 Hz in the local field potential's power spectrum, has been found and intensively studied in many brain regions. Although gamma is thought to play a critical role in processing neural information in the brain, its cognitive functions and neural mechanisms remain unclear or debatable. Experimental studies showed that gamma rhythms are stochastic in time and vary with visual stimuli. Recent studies further showed that multiple rhythms coexist in V1 with distinct origins in different species. While all these experimental facts are a challenge for understanding the functions of gamma in the visual cortex, there are many signs of progress in computational studies. This review summarizes and discusses studies on gamma in the visual cortex from multiple perspectives and concludes that gamma rhythms are still a mystery. Combining experimental and computational studies seems the best way forward in the future.

## Significance of gamma oscillations

Gamma rhythms (30–100 Hz) in the local field potential (LFP) are commonly found in many brain regions (Buzsáki 2009; Wang 2010), including the hippocampus (Bragin et al. 1995; Wang and Buzsáki 1996; Colgin et al. 2009; Belluscio et al. 2012; Fernández-Ruiz et al. 2021), entorhinal cortex (Chrobak and Buzsáki 1998; Quilichini et al. 2010), olfactory bulb (Adrian 1942, 1950; Neville and Haberly 2003), auditory cortex (Lakatos et al. 2005; Fujioka et al. 2009; Vianney-Rodrigues et al. 2011; Gross et al. 2013), parietal cortex (Bouyer et al. 1981; Pesaran et al. 2002; Hawellek et al. 2016), prefrontal cortex (Gregoriou et al. 2009; Benchenane et al. 2011; Colgin 2011; Kim et al. 2016), and visual cortex (Eckhorn et al. 1988; Gray and Singer 1989a; Gray et al. 1989; Frien et al. 1994; Kreiter and Singer 1996; Friedman-Hill et al. 2000; Maldonado et al. 2000; Hermes et al. 2015). It has been proposed that gamma-band activity may play an essential role in normal cognitive processes (Eckhorn et al. 1988; Gray et al. 1989; Singer and Gray 1995; Draguhn and Buzsáki 2004; Henrie and Shapley 2005; Fries 2005, 2009; Fries et al. 2007; Wang 2010; Buzśaki and Wang 2012) such as learning (Bauer et al. 2007), memory (Pesaran et al. 2002; Kucewicz et al. 2017), and attention (Fries et al. 2001; Jensen et al. 2007; Womelsdorf and Fries 2007; Vinck et al. 2013). Abnormal gamma rhythms are associated with mental disease (Uhlhaas and Singer 2006, 2010, 2012; Gonzalez-Burgos and Lewis 2012; Lewis et al. 2012).

Gamma-band activity was first discovered in the cat olfactory bulb (Adrian 1942, 1950). Then years later the presence of gamma activity in the primary visual cortex, V1, started drawing attention, first in cat (Eckhorn et al. 1988; Gray and Singer 1989b; Gray et al. 1989; Gray and Di Prisco 1997; Samonds and Bonds 2005), then in macaque monkey (Frien et al. 1994; Kreiter and Singer 1996; Friedman-Hill et al. 2000; Maldonado et al. 2000; Lima et al. 2010; Ray and Maunsell 2011; Jia and Kohn 2011; Womelsdorf et al. 2012; Xing et al. 2012b; Roberts et al. 2013; Jia et al. 2013a; Van Kerkoerle et al. 2014; Murty et al. 2018; Peter et al. 2019; Onorato et al. 2020) and in humans (Tallon-Baudry and Bertrand 1999; Canolty et al. 2006; Hermes et al. 2015; Michalareas et al. 2016; Kucewicz et al. 2017). Rodents (Cardin et al. 2005, 2009; Saleem et al. 2017; Storchi et al. 2017; Welle and Contreras 2017) and insects (Kirschfeld 1992; Grabowska et al. 2020) also became animal models for understanding the neural mechanisms of gamma-band activity.

**Fig. 1:**
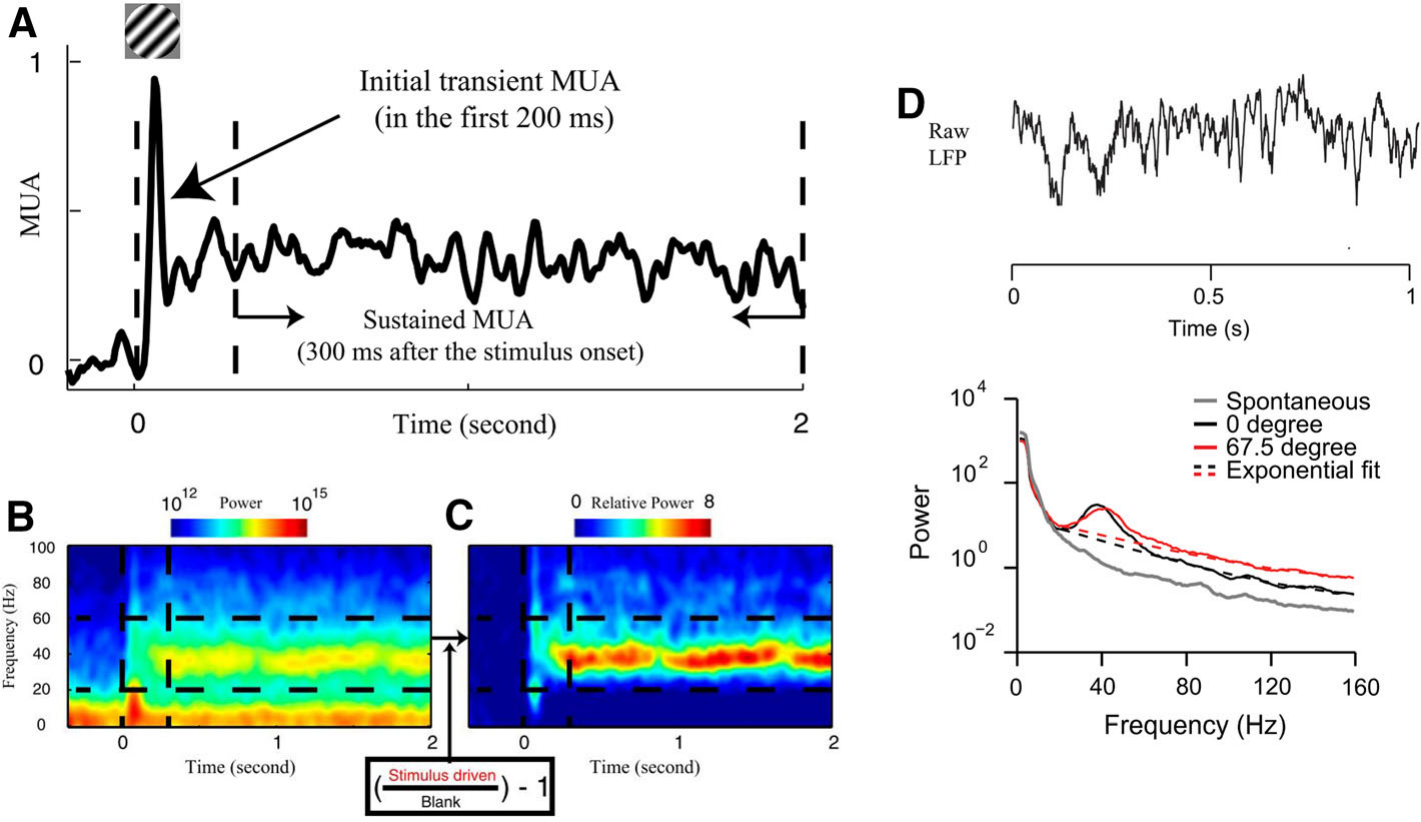
Stimulus induced narrow-band and broad-band gamma activity. **A**–**C** are replotted based on Xing et al. (2012a). **A** shows the multiunit activity to the visual stimulus (drifting grating) for an example site. The response over the first 0.2 s after stimulus onset was termed the initial transient response. The response from 0.3 to 2 s after stimulus onset (time 0) was taken to be the sustained response. **B** illustrates the color-coded spectrogram (power spectrum vs time) of the Local Field Potential (LFP) to the drifting grating stimulus. The sustained power is induced after stimulus onset (time 0 marked by the first vertical dashed line) in the frequency band between 20 and 60 Hz (marked by two horizontal dashed lines). **C** shows the relative power spectrum which is normalized by power at each frequency during the blank period. **D** is replotted based on Jia et al. (2011). The top panel of **D** shows a single epoch example of the raw LFP and the bottom panel shows the power spectra of LFPs from one site for two orientations (red and black lines) and spontaneous activity (gray lines). The gamma bump around 40 Hz is defined as the narrow-band component of gamma. The broad-band component of gamma power is estimated by the exponential fit indicated with dashed lines

In the visual cortex, gamma is observed in the local field potential (LFP) as a narrow-band increase in power in the gamma frequency range (around 30–80 Hz) during the presentation of a visual stimulus (Fig. 1A–C) (Gray and Singer 1989a; Castelo-Branco et al. 1998; Fries et al. 2007; Xing et al. 2012a; Jia et al. 2013b). The bell-shaped bump (narrow-band gamma) in the power spectrum is defined as a gamma rhythm. In the LFP, a broad-band power elevation over a wide frequency range around the gamma band (Fig. 1D) (Henrie and Shapley 2005; Yuval-Greenberg et al. 2008; Jia et al. 2011; Ray and Maunsell 2011) is a different component, which behaves similarly to spiking activity (Jia et al. 2011; Ray and Maunsell 2011). In the following sections of this paper, we focus on studies of narrow-band gamma in the visual cortex of cat and monkey, the two animal models in which studies and theories of gamma rhythms were initiated.

## The functions of gamma oscillation are controversial

There have been many speculations about the function of gamma-band activity. For example, gamma synchronization may contribute to solving the ‘binding problem’ (Singer and Gray 1995; Singer 1999a). The activity of neurons could be grouped together dynamically through synchrony associated with perceptual Gestalt principles (Milner 1974; Engel et al. 1992; von der Malsburg 1994). Engaging in an oscillation could also increase the saliency of neuronal signals (Gray and McCormick 1996) such as the relationship between orientation discrimination performance and gamma band activity (Edden et al. 2009). In V1, orientation selectivity was modulated by the gamma phase (Womelsdorf et al. 2012) and the changes of orientation preference of neuronal populations in visual cortex occurred if stimuli induced synchronized responses oscillating at gamma band frequencies (Galuske et al. 2019).

The early studies on cats (Eckhorn et al. 1988; Gray and Singer 1989a; Gray et al. 1989; Gray and Di Prisco 1997) provided evidence that global features (such as contour information, or motion information at different visual locations that belonged to the same object) of visual stimuli were associated with robust synchronization of responses in V1. These observations were then taken as support for the binding-by-synchrony (BBS) hypothesis (Milner 1974; Grossberg 1976; von der Malsburg 1994), that is, that binding/integration of information for a visual object might be through synchronization by gamma rhythms in V1 (Singer 1999b, 2009; Uhlhaas and Singer 2006, 2010, 2012). However, the BBS hypothesis has been challenged by the proposal that gamma is an epiphenomenon that is irrelevant for information processing (Shadlen and Movshon 1999). Furthermore, experimental data indicate that the peak frequency in the gamma band varies across visual cortical loci when they are activated by the same visual object (Ray and Maunsell 2010). Although there are some possible explanations of these problems for the BBS hypothesis in a follow-up study (Singer 2021), the controversy about the BBS hypothesis continues.

Several years after the BBS hypothesis was proposed came the 'communication-through-coherence' (CTC) hypothesis (Fries 2005, 2009). The CTC hypothesis proposed that gamma rhythms facilitate neuronal communication between cortical regions by neuronal coherence in the gamma-band and the effectiveness of long-distance communication depends on the relative gamma phase (zero phase) between the spikes and the LFP. Similar to the situation with the BBS hypothesis, the CTC hypothesis was both supported (Womelsdorf et al. 2007; Gregoriou et al. 2009) and opposed (Jia et al. 2013a, Akam and Kullmann 2012). An updated version of the CTC hypothesis (Fries 2015) proposed that entrainment with delay was the mechanism that sets up phase relations subserving CTC. The updated CTC proposed also that the way rhythmic synchronization modulates excitability is not simply by linear filtering of a sinusoidal drive, but with nonlinear models composed of spiking excitatory and inhibitory neurons (Borgers and Kopell 2008; Gielen et al. 2010). At present, more studies support the CTC hypothesis than oppose it, but more experiments and theories are needed to verify it in the future.

Both the BBS and CTC hypotheses implied that gamma activity performs important functions for perception or cognition. However, many studies in recent years do not support the idea that gamma rhythms in the visual cortex have specific visual functions (Singer 2018). Instead, many people think that gamma-band activity is a by-product of neuronal activity in the cortical network (Shadlen and Movshon 1999; Thiele and Stoner 2003; Roelfsema et al. 2004; Henrie and Shapley 2005; Ray and Maunsell 2010; Chariker et al. 2016, 2018) or in some cases gamma may be an artifact (Yuval-Greenberg et al. 2008). The arguments against the idea that synchronized oscillations have a functional role came from empirical studies on response properties of gamma rhythms, including studies of stimulus-dependence, stochastic dynamics, and differences of gamma-band activity in different species.

## What visual information does gamma oscillation represent?

**Fig. 2:**
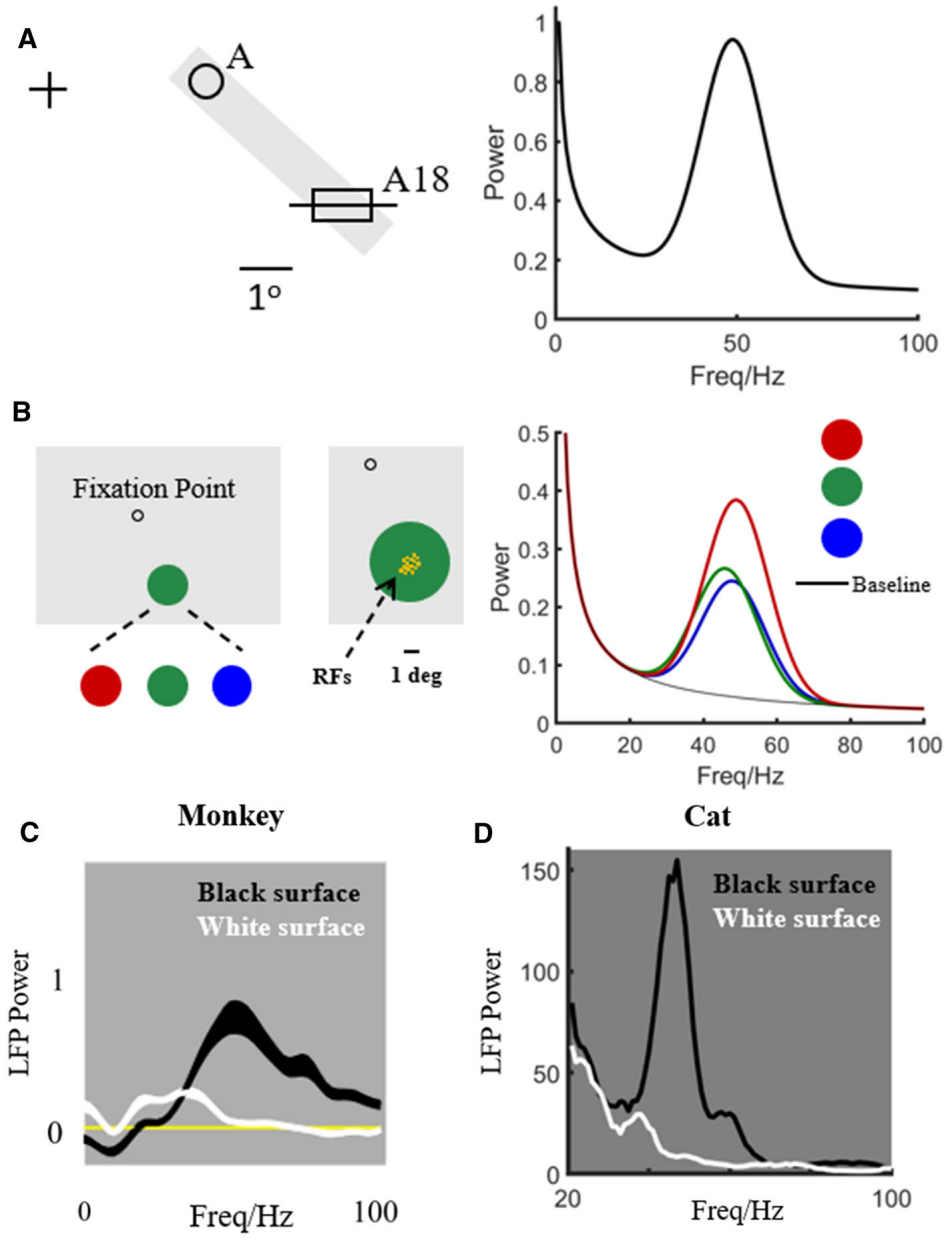
Gamma oscillation induced by surface or object stimuli. A is replotted based on Castelo-Branco et al. (1998). The bar stimulus evokes strong oscillatory responses at 49 Hz in Area 18 of the cat, as indicated by the oscillatory spectrum of spike times in the righthand panel. B is replotted based on Peter et al. (2019). It shows average LFP power spectra for different chromatic conditions. C is replotted based on Xing et al. (2014). It shows average LFP power spectra to black and white stimuli in layer 4C of V1. D shows unpublished data from Dajun Xing’s lab, which shows average LFP power spectra to black and white stimuli in cat V1

Although gamma has been thought important for visual processing, what is represented by gamma is still unclear. Some previous studies showed that gamma is strongly induced by thin bars or face contours (Fig. 2A) (Gray and Singer 1989b; Singer and Gray 1995; Neuenschwander and Singer 1996; Castelo-Branco et al. 1998; Uhlhaas and Singer 2006, 2010, 2012). Such results suggested that gamma could represent a visual object or the contour of a visible object. But other studies demonstrated that the surfaces of visual stimuli also induce strong gamma-synchronous responses in V1 (Fig. 2B) (Gail et al. 2000; Xing et al. 2010, 2014a; Peter et al. 2019), suggesting that the gamma rhythm could represent surface information (Peter et al. 2019). Whether gamma represents a visual object's contour or its surface is still unclear.

The surface preference of gamma band activity can be modified by surface properties. For instance, gamma peaks in the power spectrum of the LFP were strongly induced by a black surface, but the gamma peak was weak or absent for a white surface in macaque V1 (Xing et al. 2014) (Fig. 2C) or cat V1 (Fig. 2D, unpublished data from Dajun Xing’s lab). A similar result could also be seen in the color preference of gamma; gamma prefers red surfaces more than surfaces of other colors (Shirhatti and Ray 2018; Peter et al. 2019). If gamma represents visual surfaces, why there is such a strong luminance/color preference? One possible answer is that it could be simply a consequence of different cone contrast–red versus gray higher in cone contrast than green versus gray, for instance. But the color preference still needs to be confirmed and explored more deeply in future experiments.

The uncertainty about gamma's function for information representation may be due to the fact that we don't understand fully the mechanisms for the stimulus dependence of gamma band rhythms in the visual cortex. Previous studies have shown that gamma depends on different stimulus characteristics, such as luminance/color (Swettenham et al. 2013; Xing et al. 2014; Saleem et al. 2017; Storchi et al. 2017; Shirhatti and Ray 2018; Peter et al. 2019), contrast (Henrie and Shapley 2005; Ray and Maunsell 2010; Jia et al. 2013b), orientation (Siegel and König 2003; Kayser and Ko 2004; Cardin et al. 2005; Berens et al. 2008; Womelsdorf et al. 2012; Han et al. 2020), temporal frequency (Jia et al. 2011; Murty et al. 2018), stimulus size (Bauer et al. 1995; Gieselmann and Thiele 2008; Jia et al. 2011, 2013b; Ray and Maunsell 2011). Furthermore, the complexity of visual stimuli modulates the strength of cortical gamma activity. Gamma band power is reduced when the visual stimulus includes a noise masking grating (Zhou et al. 2008; Jia and Kohn 2011; Jia et al. 2013a), and in natural images(Kayser et al. 2003; Kayser and Ko 2004; Hermes et al. 2015), and when the visual stimulus consists of superimposed gratings (Lima et al. 2010; Bartolo et al. 2011; Wang et al. 2021). In the last section of our review, we will discuss progress in understanding the stimulus-dependence of gamma rhythms.

## How does gamma band activity help information integration?

In addition to visual information representation, the relationship between gamma rhythms and visual information integration is also unclear. Spike activity, which carries visual information, has been found to lock to the phase of the gamma rhythm (Schoffelen et al. 2005; Fries et al. 2007; Fries 2015). The strong phase-locking between visually-evoked signals and gamma suggests that different kinds of visual information might be communicated according to their gamma phase. Perhaps phase-locking together with the CTC hypothesis (Fries 2005, 2009, 2015) could explain how different visual signals about visual objects could be integrated in downstream cortical regions.

**Fig. 3:**
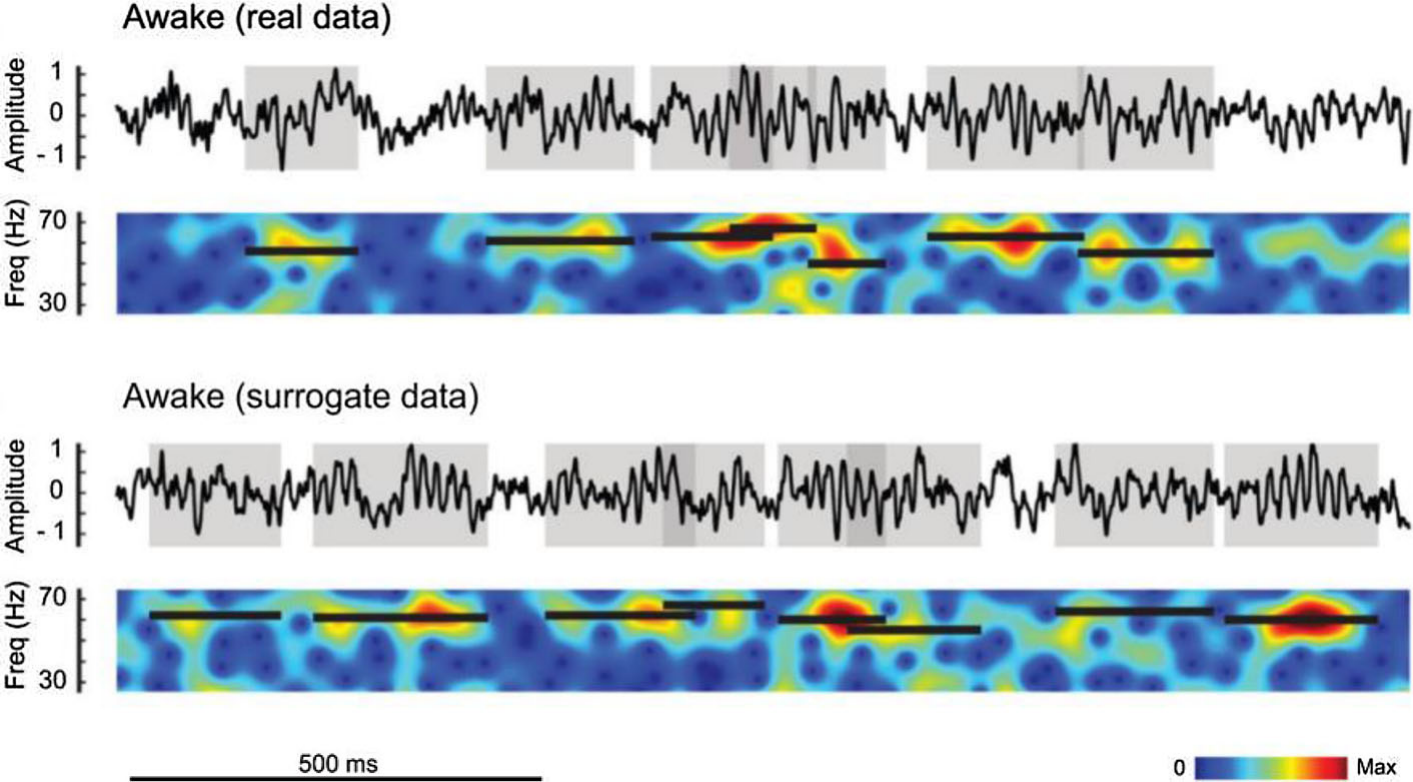
Stochastic generation of gamma in awake and anesthetized states. The figure is replotted based on Xing et al. (2012b). The top panel shows the LFP recorded from an awake monkey after stimulus onset and in the panel below it is its spectrogram. The gray shaded areas of the LFP are gamma-band bursts. Each black bar in the spectrogram marks the peak frequency (y-axis) and the duration of each gamma-band burst

However how the visual system coordinates/integrates gamma information in time and phase is not fully understood. Some theoretical work has used oscillator models to understand gamma rhythm (Börgers 2017). A natural consequence of the oscillator theories is the concept of gamma as a clock signal for synchronizing brain information. However, experimental studies found that gamma rhythms in V1 are not oscillatory but instead are stochastic in time (Burns et al. 2010, 2011; Xing et al. 2012a) (Fig. 3, but see Subhash Chandran et al. 2018). There are rapid changes of frequency and only short durations of consistent phase and power elevation (Also see Feingold et al. 2015 for that of beta rhythm in motor cortex and Bastos et al. 2018 for gamma bursting in frontal cortex). The non-oscillatory nature of gamma was shown theoretically to cause more CTC (Saraf and Young 2021) suggesting that instaneous synchronization, instead of sustained oscillation, is more important for information integration (Xing et al. 2012a). Although there are studies (Jia et al. 2013a; Roberts et al. 2013) that showed that gamma's stochastic properties are consistent and coordinated between monkey V1 and V2, we still don't know the exact way that the brain integrates the information carried by gamma in time. We need to rethink how gamma rhythms are generated and how gamma rhythms might aid information processing in the brain.

Besides the randomness of gamma in time, the gamma rhythm is also variable in space. Ray and Maunsell (2010) showed that frequencies of gamma rhythms could be continuously different as a function of location in visual space, even if the different locations belong to the same visual object. The frequency difference for gamma in space is somewhat puzzling; if specific visual information appears in a fixed phase for all different frequencies, then the information carried by these frequencies will appear at different times; if the visual system synchronizes the information at the same time, then the information will appear in different phases for various frequencies.

**Fig. 4:**
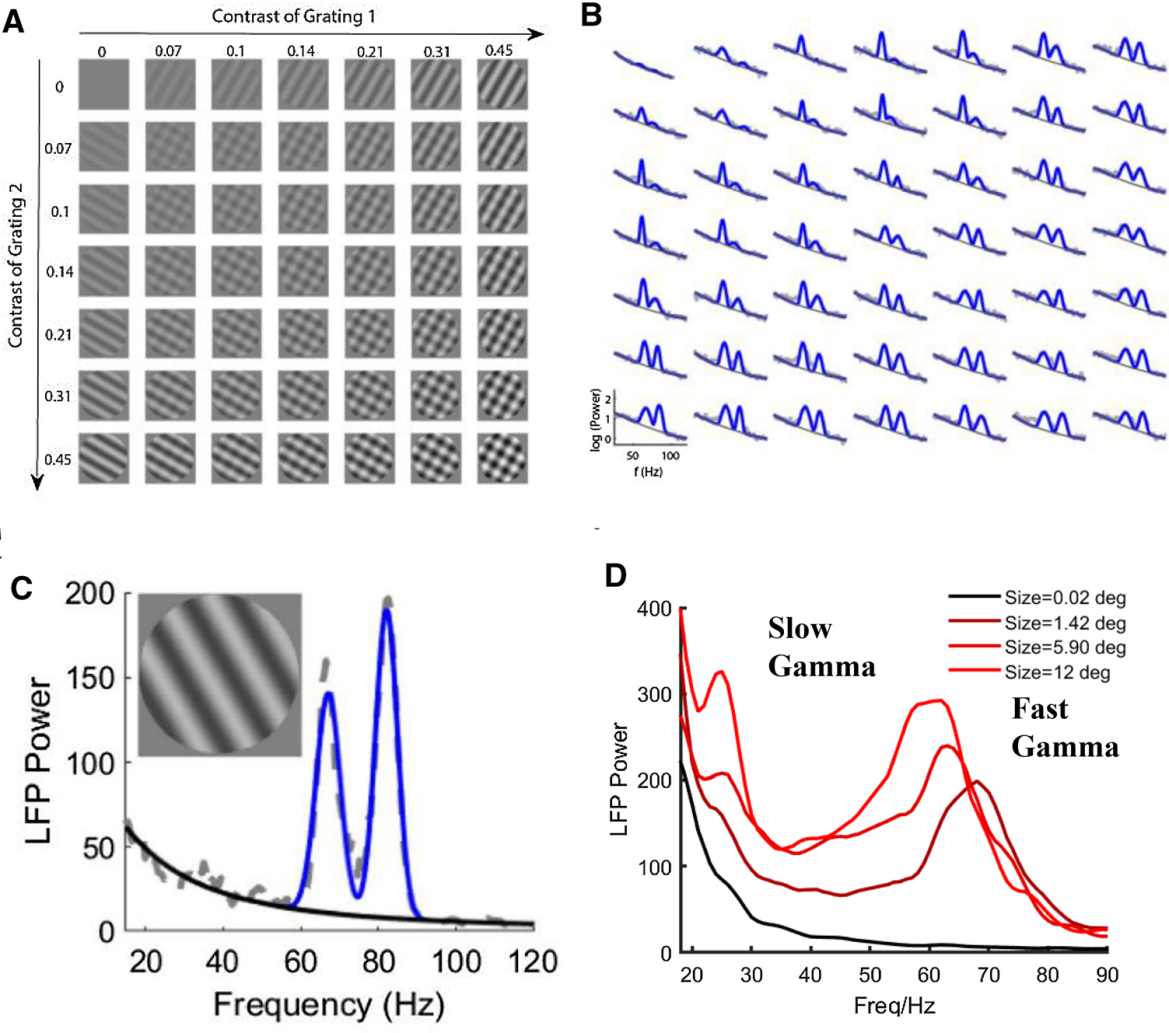
Experimental evidence for multiple gamma oscillations in the visual cortex. **A**–**C** is replotted based on Wang et al. (2021). **A** and **B** show the response matrix driven by plaid for an example site. A presents various plaids formed by the linear summation of two orthogonal drift gratings with varying contrast. These stimuli (size, 38°; spatial frequency, 0.05 cycle/deg) were presented for 2 s with a time–frequency of 2 Hz and were repeated 10 times. **B** shows the trial averaged (*n* = 10) LFP power spectrum (gray dots) in response to corresponding stimuli in **A**. Two narrow-band gamma oscillations (blue curve) and the baseline (dark gray line) were estimated through a spectrum fitting procedure. **C** shows the LFP power spectra have two distinct narrow-band gamma oscillations induced by a grating in cat V1. **D** shows the LFP power spectra in awaked macaque V1 that have two distinct narrow-band gamma oscillations induced by gratings with different size, which is the unpublished data from Dajun Xing’s lab

Most studies on gamma band activity have reported a single gamma frequency bump in monkey V1. Interestingly, two gamma peaks in the frequency spectrum have been previously reported in the visual cortex of humans (Kucewicz et al. 2017), cats (Fig. 4A–C) (Castelo-Branco et al. 1998; Bharmauria et al. 2016; Han et al. 2020; Wang et al. 2021), monkeys (Murty et al. 2018 and Fig. 4D is unpublished data for awake monkey V1 from Dajun Xing's lab) and rats (Oke et al. 2010). The finding of multiple gammas generates more questions about gamma's function. What kind of visual information is carried by two distinct gamma peaks, and what is the relationship between the two gamma peaks? How do downstream cortical regions integrate information carried by multiple gammas? All these questions have not been answered fully.

## Do gamma rhythms serve a canonical function?

If gamma band activity has essential functions in cognition and perception, one should expect that its importance will lead to conservation of brain functions and circuits that generate gamma in different species throughout biological evolution. However, this seems not to be the case; instead, recent studies have shown that gamma band activity in different species' visual cortices are different in response properties and neural origins.

**Fig. 5:**
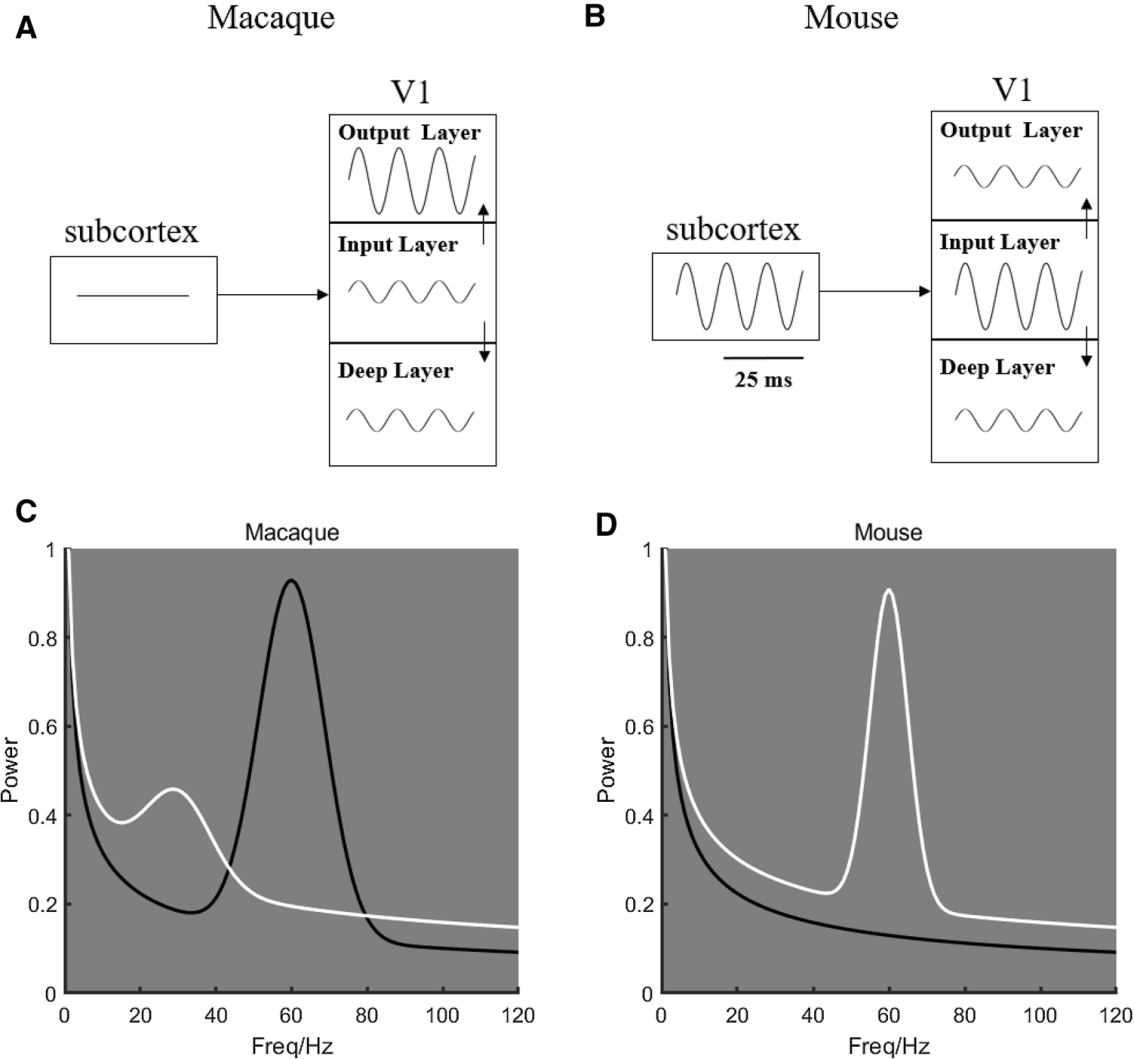
Species difference on generation mechanism and functions of gamma rhythms. **A** is plotted based on the main result in Bastos et al. (2014) which shows the cortical mechanism of the gamma rhythm. **A** is plotted based on the main result in Saleem et al. (2017) which shows the subcortical mechanism of the gamma rhythm. **C** is replotted based on Xing et al. (2014). It shows the average power spectra (LFP) to black and white stimuli in macaque V1. **D** is replotted based on Storchi et al. (2017) and Saleem et al. (2017). It shows the average LFP power spectra during uniform light conditions (white) or complete darkness (black)

Bastos et al. (2014), studying macaque monkeys, found gamma in V1 (specifically in V1 superficial layers Spaak et al. 2012; Xing et al. 2012b; Roberts et al. 2013; Van Kerkoerle et al. 2014) but not in the LGN and concluded that a visually-induced gamma rhythm is an emergent property in the cortex (Fig. 5A). However, for mice, studies have shown that gamma is generated in subcortical regions, including LGN (Saleem et al. 2017; Schneider et al. 2021) and retina (Storchi et al. 2017) (Fig. 5B). Studying cat visual cortex, Castelo-Branco et al., (1998) found two narrow-band gamma peaks. Their results indicated that one is generated at the cortical level, and the other one is of subcortical origin. More importantly, the response properties of gamma rhythms are also species-specific. In macaque, Xing et al. (2014) found that the peak in the gamma band preferred dark stimuli (Figs. 2D, 5C for macaque data; also see Fig. 2D for unpublished data in cats from Dajun Xing’s lab), while in mouse V1, the LFP gamma activity was strongly induced by lightness instead of darkness (Fig. 5D) (Saleem et al. 2017; Storchi et al. 2017). The species differences are not consistent with a canonical role of gamma in cognition and perception.

## Neural mechanism of gamma rhythms revealed by computational models

We have to admit that we don't fully understand the function, or functions, of gamma rhythms given all the puzzles and controversies reviewed above. This is primarily due to the fact that we also don't know the neural mechanisms for generating and modulating gamma. With advanced experimental techniques (intracellular recordings, optogenetics, etc.), mouse studies have revealed excitatory and inhibitory contributions to gamma (Cardin et al. 2009; Veit et al. 2017). But so far these techniques cannot be fully applied in primate studies. The species difference for gamma properties suggests a need for separate studies on different species. An alternative way to understand gamma at the circuit level for primates is to construct dynamic models of neural networks combining experimental data.

**Fig. 6:**
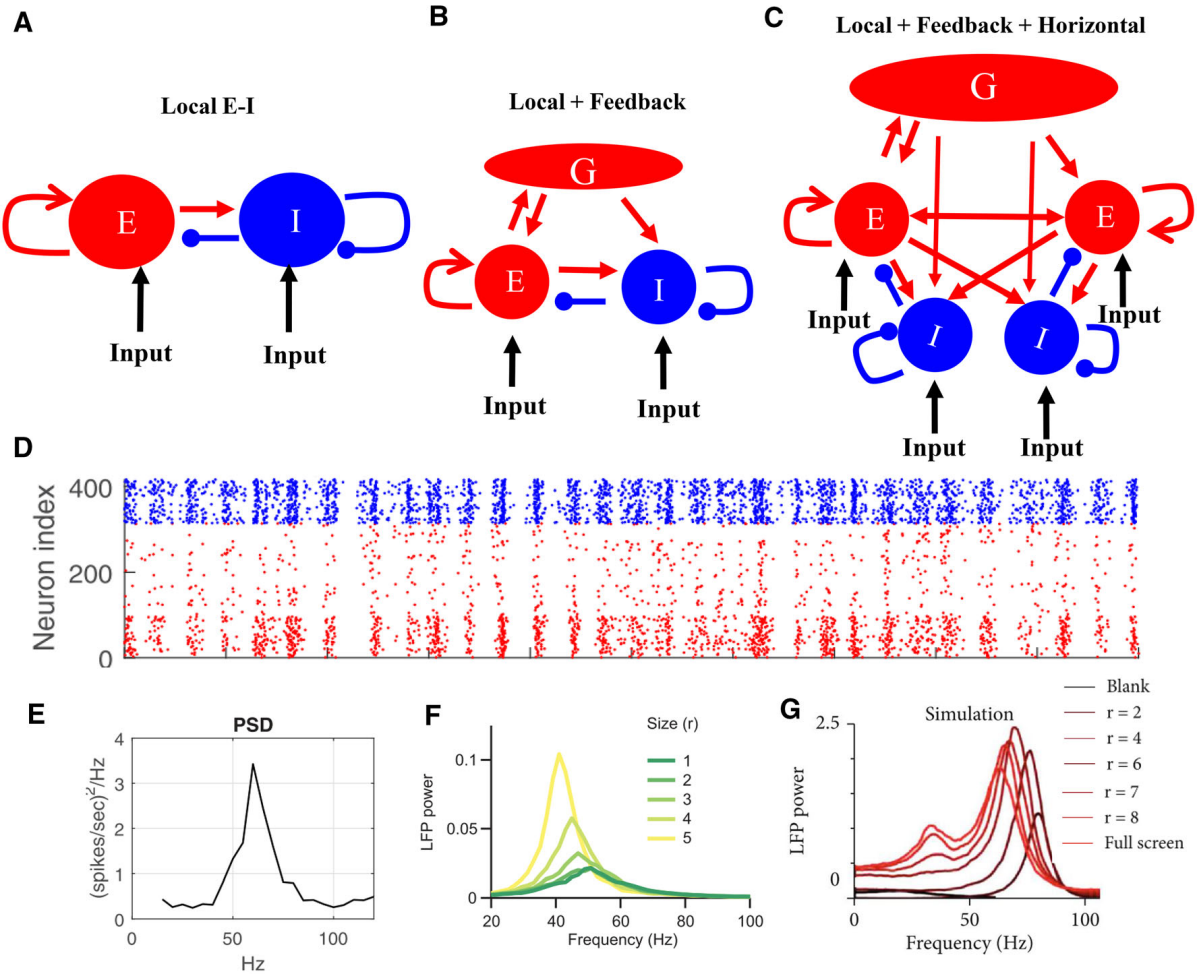
Architectures and connectivity patterns of models for generating narrow-band gamma rhythms. **A** shows the PING model's basic framework (mutually connected pyramidal cell and interneuron). **B** shows a model based on the PING model in **A**, but it has an additional feedback component, which is driven by the E neurons and provided excitatory feedback to both E and I (Jia et al. 2013a, b). **C** shows a model based on the model in **B**, with added horizontal connections (HC) in V1 (Han et al. 2021). **D** (replotted based on Chariker et al. 2018) shows the raster of spikes generated in a large scale spiking neural network (in a patch of 400 E- and I- neurons in a cortical hyper-column). The spiking neuron model is also able to generate gamma band peaks with stochastic inputs, which is shown in the Power Spectral Density (PSD) plot in **E**. **F** is replotted based on Jia et al. (2013a, b). It shows the simulated power spectra of LFP for gratings of different sizes based on the model shown in **B**. **G** is replotted based on Han et al. (2021). It shows power spectra of the LFP induced by stimuli at different sizes in the model shown in **C**

Computational models with interaction of excitation and inhibition (Fig. 6A) (Xing et al. 2012a; Chariker et al. 2016, 2018; Mejias et al. 2016) (Fig. 6DE) have made progress in understanding stochastic and stimulus-dependent properties of gamma. The gamma peaks are strongly influenced by different types of neural circuits. Local circuit models, have successfully achieved the stochastic properties observed in experiments (Xing et al. 2012a; Chariker et al. 2018). Furthermore, Jia and colleague (2013b) introduced feedback connections in the V1 model (Fig. 6B) and explained many observations concerning the changes of gamma power and frequency with different stimuli, including stimulus contrast, size, orientation, and noise-masking stimuli (Jia et al. 2013b) (Fig. 6F). More recently, Han et al. (2021) introduced horizontal connections (HC) into a large-scale V1 model (Fig. 6C). They found that HC could generate a new gamma around 30 Hz (slow gamma) which is different from fast gamma (around 50–60 Hz)(Fig. 6G). The two gamma band peaks that emerge in the Han et al. (2021) model are highly consistent with experimental findings for two distinct gammas in macaque V1(Fig. 4D).

Up to now, computational models have provided possible mechanisms for various gamma band phenomena in visual cortex, but there are still two main shortcomings. The first is that the predicted effects on gamma rhythms from feedback and horizontal neural circuits in the model should be further confirmed by experiments. Another one is that most of the models to explain stimulus dependence and stochastic property are at the mean-field level. Spiking neuron models (like Chariker et al. 2018) are closer to the actual response of neurons and will play a greater role in the future.

## Concluding Remarks

Although the cognitive functions of gamma band peaks in cortical population activity are still unclear, it is apparent that gamma is related to multiple neural circuits, suggested by computational studies. As an essential feature for a complex dynamic system (the brain), gamma band peaks are crucial experimental data that should help us to understand neural circuits underlying brain functions. From both experimental and computational sides, more work on gamma band activity is needed for understanding its role in normal cognitive functions and abnormal mental states of the brain.
